# ^18^F-FDG PET/CT-based metabolic metrics in recurrent tumors of ovarian clear cell carcinoma and their prognostic implications

**DOI:** 10.1186/s12885-019-5441-7

**Published:** 2019-03-13

**Authors:** Shuang Ye, Shuai Liu, Libing Xiang, Xiaohua Wu, Huijuan Yang

**Affiliations:** 1 Department of Gynecologic Oncology, Fudan University Shanghai Cancer Center, Shanghai, 200032 China; 2 Department of Nuclear Medicine, Fudan University Shanghai Cancer Center, Shanghai, China; 3 Department of Oncology, Shanghai Medical College, Fudan University, Shanghai, 200032 China

**Keywords:** Ovarian neoplasms, Clear cell carcinoma, Positron emission tomography computed tomography, Recurrence, Survival

## Abstract

**Background:**

Glucose metabolism has been suggested as a therapeutic target in ovarian clear cell carcinoma (CCC). We attempted to clarify ^18^F-FDG PET/CT-based metabolic metrics in the recurrent ovarian CCC patients and their prognostic values.

**Methods:**

Quantitative metabolic parameters included maximum standardized uptake value (SUVmax), metabolic tumor volume (MTV) and total lesion glycolysis (TLG). Two different methods were employed for defining the threshold SUV to delineate MTV: 1) SUV of 2.5 (designated as MTV); 2) a fixed ratio including 40% (MTV40), 50% (MTV50) and 60% (MTV60) of SUVmax. The Kaplan-Meier model and Cox regression were used in survival analysis.

**Results:**

Among the 35 patients, platinum-resistant recurrence accounted for 34.3% and the median progression-free survival was 13 months (range, 2–135). Fifteen (42.9%) patients presented with single tumor recurrence, while 51 recurrent lesions were identified, with the most common sites in pelvis (29.4%), followed by lymph node metastases (19.6%) and peritoneal carcinomatosis (15.7%). Except four patients with FDG-inavid tumor, the median SUVmax of the 31 patients with high glucose metabolic activity was 7.10 (range, 3.00–20.60). After a median follow-up of 36.5 months (range, 7–155), 22 patients (64.7%) were dead from disease. The median post-relapse survival (PRS) was 17 months (range, 4–126). Platinum-resistant recurrence, peritoneal carcinomatosis and high TLG60 proved to be negative predicators of overall survival after multivariate analysis.

**Conclusions:**

TLG60, platinum-resistant recurrence and peritoneal carcinomatosis were independent negative predicators of overall survival. Whether patients with higher TLG60 required more aggressive treatment warranted further study.

## Background

Ovarian clear cell carcinoma (CCC) is a distinct histologic subtype, accounting for 5–25% of all epithelial ovarian cancer depending on geographic location [1, 2]. Ovarian CCC represents a great challenge due to its disease aggressiveness and chemotherapy resistance [1, 2]. To be worse, recurrent ovarian CCC is particularly chemoresistant [3]. The response rate to systemic therapy is less than 10% even in the setting of platinum-sensitive recurrence [3]. In two recent publications, biologic agents (sunitinib and cabozantinib) demonstrated minimal activity in the second- and third-line treatment of persistent or recurrent ovarian CCC [4, 5]. Different from other histologic subtype, ovarian CCC has its unique glucose metabolic activity, which might have a future clinical implication of targeting cancer-specific metabolism [6–8]. Thus, clarifying the features of metabolic activity and recurrence pattern in ovarian CCC might help to guide new treatment strategies tailored to ovarian CCC.

As a molecular imaging technique, ^18^F-fluorodeoxyglucose positron emission tomography/computed tomography (^18^F-FDG PET/CT) is used to evaluate glucose metabolism and tumor distribution in different kinds of cancers including gynecologic malignancy [9]. It has been widely employed in detecting recurrence and restaging in ovarian cancer patients [10]. Several publications have examined the role of ^18^F-FDG PET/CT quantitative metrics in ovarian cancer, usually lumping different histologic subtypes together [10–19]. PET/CT-based metabolic parameters include maximum standardized uptake value (SUVmax), metabolic tumor volume (MTV) and total lesion glycolysis (TLG). MTV refers to the estimated volume of tumor with increased tracer uptake while TLG is an estimation of summed metabolic activity inside MTV. In a previous study, metabolic variables at the time of first recurrence were proved to be significant predictors of post-relapse survival (PRS) in ovarian cancer patients [10]. Nevertheless, there has been no publication focusing on the clinical utility of PET/CT-based metabolic variables in patients with recurrent ovarian CCC.

The objective of the present study was to delineate the recurrence pattern of ovarian CCC. A secondary aim was to investigate the possible roles of PET/CT-based metabolic variables in survival prediction in patients with relapsed ovarian CCC.

## Methods

### Patients

After obtaining the approval by the institutional review board (SCCIRB-090371-2), we included all the ovarian CCC patients who received ^18^F-FDG PET/CT scan at first recurrence from 2008 to 2017. Patients had to fulfill the following criteria to be included into the study: 1) histologically confirmed diagnosis of ovarian CCC. Patients initially treated in outside hospital were required to have pathology consultation in our institution as a routine practice. 2) ^18^F-FDG PET/CT performed in our institution at the first relapse. Clinicopathological features were reviewed and retrieved. Data collection included age at first diagnosis, International Federation of Gynecology and Obstetrics (FIGO) stage [20], platinum-free interval, serum cancer antigen 125 (CA 125) and CA199 level at relapse, recurrent tumor location, peritoneal carcinomatosis, type of second-line treatment, and tumor status at the date of last contact. Patients were considered to have platinum-sensitive disease if the interval time was > 6 months from the completion of the last platinum based chemotherapy to disease recurrence. Progression-free survival (PFS) and overall survival (OS) was defined as the time interval from the date of the primary surgery to the date of first recurrence and death or last contact, respectively. Post-relapse survival (PRS) was calculated as the time interval from the date of diagnosis of relapsed disease to the date of death or last contact. Patients with incomplete follow-up information were excluded from the survival analysis. A total of 35 patients were included into the study.

### ^18^F-FDG PET/CT protocol and image analysis

The ^18^F-FDG PET/CT protocol was introduced specifically in our previous publication [18]. A gynecologic oncology dedicated nuclear medicine physician (Dr. Liu) interpreted the images retrospectively, who was blind to the clinicopathologic information. Standardized uptake value (SUV) is defined as [decay corrected activity (kBq) per milliliter of tissue volume]/[injected 18 F-FDG activity (kBq) per gram of body mass]. In line with our previous publication, SUVmax was calculated by placing a spheroid-shaped volume of interest within the primary ovarian tumor. MTV and SUVaverage were evaluated by drawing a contour of the ovarian tumor large enough to encase the tumor in the axial, coronal and sagittal section. In the current work, two methods were used for defining threshold SUV to delineate MTV: 1) a threshold of SUV of 2.5 (designated as MTV); 2) a fixed ratio including 40% (MTV40), 50% (MTV50) and 60% (MTV60) of SUVmax. The boundaries of voxels exceeding the defined threshold were automatically produced and those presenting an SUV greater than the threshold were incorporated to MTV measurement. TLG was calculated as MTV × SUVaverage. The highest SUVmax of all recurrent lesions was determined as SUVmax for one patient, while volumetric metrics (MTV/TLG) were required by summing up all the values of relapsed tumors. PET/CT-based metabolic variables were assessed in 31 patients, given that four (11.4%) presented with low glucose-uptake tumor.

### Statistical analysis

Statistical Package for Social Science (SPSS) (Version 20.0, SPSS, Inc., Chicago, IL, USA) and GraphPad Prism (Version 6.0, GraphPad Software, Inc., La Jolla, CA, USA) were used for the analyses. Clinicopathological variables and PET/CT metrics were presented using descriptive statistics. Medians and ranges were used for continuous variables, while proportions were used for categorical data. Survival time was calculated using the Kaplan-Meier model, whereas Cox regression was performed for multivariate analysis. Variables with statistical significance on univariate analysis were included in the multivariate one. To evaluate the relationship between quantitative PET/CT variables and survival outcome, patients were dichotomized based on the median number. All *P* values reported were two tailed, and *P* < 0.05 was considered statistically significant.

## Result

### Patient characteristics and patterns of recurrence

Table 1 shows the clinicopathological characteristics of the 35 patients included in the study. Nearly a half of the patients (44.8%) presented with early-stage disease (FIGO I + II) at initial diagnosis. Overall, platinum-resistant recurrence accounted for 34.3%. The median progression-free survival was 13 months (range, 2–135). At recurrence, normal serum level of CA 125 and CA199 was noted in 17 (48.6%) and 22 (62.3%) patients, respectively. Fifteen (42.9%) patients presented with single tumor recurrence. In terms of treatment, 27 patients (77.1%) received secondary cytoreduction surgery (SCS) in our institution. One patient lost follow-up after SCS, thus excluded from the survival analysis. After a median follow-up time of 36.5 months (range, 7–155), 22 patients (64.7%) were dead from disease and 12 patients (35.3%) were still alive with disease.

**Table 1:** Patient characteristics (*n* = 35)

Variables	
Age at initial diagnosis (years), median (range)	51 (28–77)
FIGO stage at diagnosis (%)^a^	
Early (I + II)	13 (44.8%)
Advanced (III + IV)	16 (55.2%)
Platinum resistant recurrence (%)	12 (34.3%)
Serum CA-125 level at recurrence (U/mL), median (range)	35.1 (3.90–829.2)
Serum CA-199 level at recurrence (U/mL), median (range)	12.01 (0.5–1000)
Number of recurrent tumor lesions	
Single (%)	15 (42.9%)
Multiple (%)	20 (57.1%)
Type of treatment	
Secondary cytoreduction surgery + chemotherapy	27 (77.1%)
Chemotherapy	8 (22.9%)
Follow up time (months), median (range)^b^	36.5 (7–155)
Disease status at last follow up^b^	
Dead (%)	22 (64.7%)
Alive with disease (%)	12 (35.3%)
Progression-free survival (months), median (range)	13 (2–135)
Post-relapse survival (months), median (range)	17 (4–126)

^a^Six patients received primary surgery at outside hospital and stage was not documented

^b^One patient lost follow up after secondary debulk surgery for single recurrent tumor

A total of 51 recurrent lesions were identified in the 35 patients. The specific recurrent tumor distributions are listed in Table 2. The most common sites of disease were pelvis (*n* = 15, 29.4%), followed by lymph node metastases (*n* = 10, 19.6%) and peritoneal carcinomatosis (*n* = 8, 15.7%). There were six vaginal cuff recurrences. Parenchymal solid organ metastases were noted in lung (*n* = 2, 3.9%), liver (n = 2, 3.9%) and spleen (n = 1, 2.0%), respectively. It is worthy of mentioning that four patients (7.8%) had abdominal wall lesions.

**Table 2:** Recurrent tumor distribution (*n* = 51)

	Numbers (%)
Pelvis	
Pelvic tumor	7 (13.7%)
Vaginal cuff tumor	6 (11.8%)
Pelvic peritoneum	1 (2.0%)
Rectal tumor	1 (2.0%)
Peritoneal carcinomatosis	8 (15.7%)
Lymph node metastases	
Multiple lymph nodes	7 (13.7%)
Peri-aortic lymph node	1 (2.0%)
Retro-pancreatic lymph node	1 (2.0%)
Supra-diaphragmatic lymph node	1 (2.0%)
Lung metastasis	2 (3.9%)
Spleen metastasis	1 (2.0%)
Liver metastasis	2 (3.9%)
Hepatorenal recess tumor	3 (5.9%)
Left-side diaphragmatic tumor	1 (2.0%)
Mesocolic tumor and paracolic tumor	3 (5.9%)
Abdominal wall tumor	4 (7.8%)
Intestinal mesentery	2 (3.9%)

We were particularly interested in the relapse pattern of patients with initially early-stage disease, which are presented in Table 3. The median progression-free survival was 20 months (range, 6–108). Overall, three (23%) patients developed platinum-resistant recurrences. Interestingly, a pelvic component of relapse was observed in more than half the patients (7/13, 53.8%) and five patients (5/13, 38.5%) had a solitary pelvic lesion. On the whole, eight patients (61.5%) had solitary lesions. The majority of the patients underwent secondary debulking surgery. The median PRS was 26.5 months (range, 6–126). The estimated 5-year survival was 59.4% for the entire cohort.

**Table 3:** Recurrence pattern of patients with early-stage disease at first diagnosis (*n* = 13)

No	Age	Stage	PFS (Months)	Platinum response	CA125 (U/mL)	Relapsed tumor	SUVmax	Second-line treatment	Status at last follow-up	PRS (Months)	OS (Months)
1	51	II	24	Sensitive	52.56	Vaginal cuff	(−)^a^	SCS + Chemo	Alive	126	150
2	47	I	48	Sensitive	3.90	Vaginal cuff	(−)^a^	SCS + Chemo	Alive	94	142
3	53	II	13	Sensitive	10.50	Pelvic tumor	7.2	SCS + Chemo	Dead	29	42
4	54	I	19	Sensitive	4.9	Lung	3.4	SCS + Chemo	Alive	95	114
5	54	I	31	Sensitive	48.60	Multiple lymph nodes	4.7	SCS + Chemo	Dead	43	74
6	53	II	6	Resistant	11.24	Abdominal wall tumor, liver, rectal tumor	13.4	SCS + Chemo	Dead	6	12
7	59	I	20	Sensitive	21.63	Multiple lymph nodes	7.9	SCS + Chemo	Dead	10	30
8	60	I	108	Sensitive	115.30	Supra-diaphragmatic lymph node, Intestinal mesentery, pelvic tumor, vaginal cuff	14.3	SCS + Chemo	Alive	36	144
9	36	I	34	Sensitive	8.31	Spleen	5.4	SCS + Chemo	Dead	24	58
10	46	I	9	Resistant	23.74	Abdominal wall tumor	9.0	SCS + Chemo	Alive	18	27
11	62	I	6	Resistant	40.65	Pelvic tumor	4.9	SCS + Chemo	Alive	13	19
12	34	II	34	Sensitive	154.30	Multiple lymph nodes, peritoneum, liver	20.6	Chemo	Alive	15	49
13	48	I	13	Sensitive	32.21	Pelvic tumor	5.0	SCS	Unknown^b^	Unknown^b^	Unknown^b^

^a^Two patients presented with low glucose-uptake tumor

^b^This patient lost follow up after second cytoreduction surgery in our hospital

Abbreviations: *PFS* Progression Free Survival, *SUVmax* Maximum Standardized Uptake Value, *PRS* Post-Relapse Survival, *OS* Overall Survival, *SCS* Secondary Cytoreduction Surgery, *Chemo* Chemotherapy

### Glucose uptake quantification metrics and prognostic implication

Among 35 patients, four presented with low glucose-uptake tumor: two vaginal cuff lesions, one pelvic tumor and one hepatorenal recess tumor. Therefore, PET/CT-based metabolic parameters were calculated for a total of 31 patients, which is presented in Table 4. The median SUVmax was 7.10 (range, 3.00–20.60). The median values of metabolic parameters were used as cut-offs for subsequent survival analysis, which is illustrated in Table 5.

**Table 4:** Metabolic parameters (*n* = 31)

Variables	
SUVmax (g/mL), median (range)	7.10 (3.00–20.60)
MTV (mL), median (range)	17.59 (0.23–477.43)
TLG (g), median (range)	88.86 (0.65–2223.38)
MTV40 (mL), median (range)	19.79 (0.46–377.37)
TLG40 (g), median (range)	87.91 (3.17–1453.47)
MTV50 (mL), median (range)	13.48 (0.34–217.36)
TLG50 (g), median (range)	64.36 (2.22–1095.26)
MTV60 (mL), median (range)	8.68 (0.23–120.34)
TLG60 (g), median (range)	38.24 (1.63–795.41)

Abbreviations: *SUVmax* Maximum Standardized Uptake Value, *MTV* Metabolic Tumor Volume, *TLG* Total Lesion Glycolysis

**Table 5:** Survival analyses

Univariate analysis						
Variables	*P* (OS)			*P* (PRS)		
Stage at initial diagnosis (Early Vs. Late)	0.006*			0.009*		
Platinum response (Sensitive Vs. Resistant)	< 0.001*			0.051		
Type of second-line treatment (SCS + chemo Vs. Chemo)	0.012*			0.021*		
Number of relapsed lesions (Single Vs. Multiple)	0.008*			0.019*		
Peritoneal carcinomatosis (No Vs. Yes)	0.001*			< 0.001*		
SUVmax (Low Vs. High)^†^	0.768			0.774		
MTV (Low Vs. High)^†^	0.083			0.128		
TLG (Low Vs. High)^†^	0.079			0.112		
MTV40 (Low Vs. High)^†^	0.141			0.091		
TLG40 (Low Vs. High)^†^	0.095			0.108		
MTV50 (Low Vs. High)^†^	0.129			0.098		
TLG50 (Low Vs. High)^†^	0.095			0.108		
MTV60 (Low Vs. High)^†^	0.129			0.098		
TLG60 (Low Vs. High)^†^	0.044*			0.031*		
Multivariate analysis						
Variables	OS			PRS		
	HR	95% CI	*P* value	HR	95% CI	*P* value
Stage at initial diagnosis	1.034	0.252–4.2	0.963	2.1	0.6–7.3	0.224
Platinum response	16.5	3.2–85.2	0.001*	/	/	/
Type of second-line treatment	1.085	0.5–24.8	0.959	0.3	0.1–5.4	0.424
Number of relapsed lesions	1.1	0.3–4.2	0.862	2.8	0.7–10.8	0.140
Peritoneal carcinomatosis	12.1	2.4–61.1	0.002*	7.7	2.2–27.3	0.002*
TLG60	4.8	1.2–18.7	0.024*	2.0	0.6–6.7	0.254

†Metabolic parameters were dichotomized using the median number as cut-off value

*P values with statistical significance were denoted by bold characters in underline

Abbreviations: *OS* Overall Survival, *PRS* Post-Relapse Survival, *SCS* Secondary cytoreduction surgery, *Chemo* Chemotherapy, *SUVmax* Maximum Standardized Uptake Value, *MTV* Metabolic Tumor Volume, *TLG* Total Lesion Glycolysis, *HR* Hazard Ratio, *CI* Confidence Interval

**Fig. 1:**
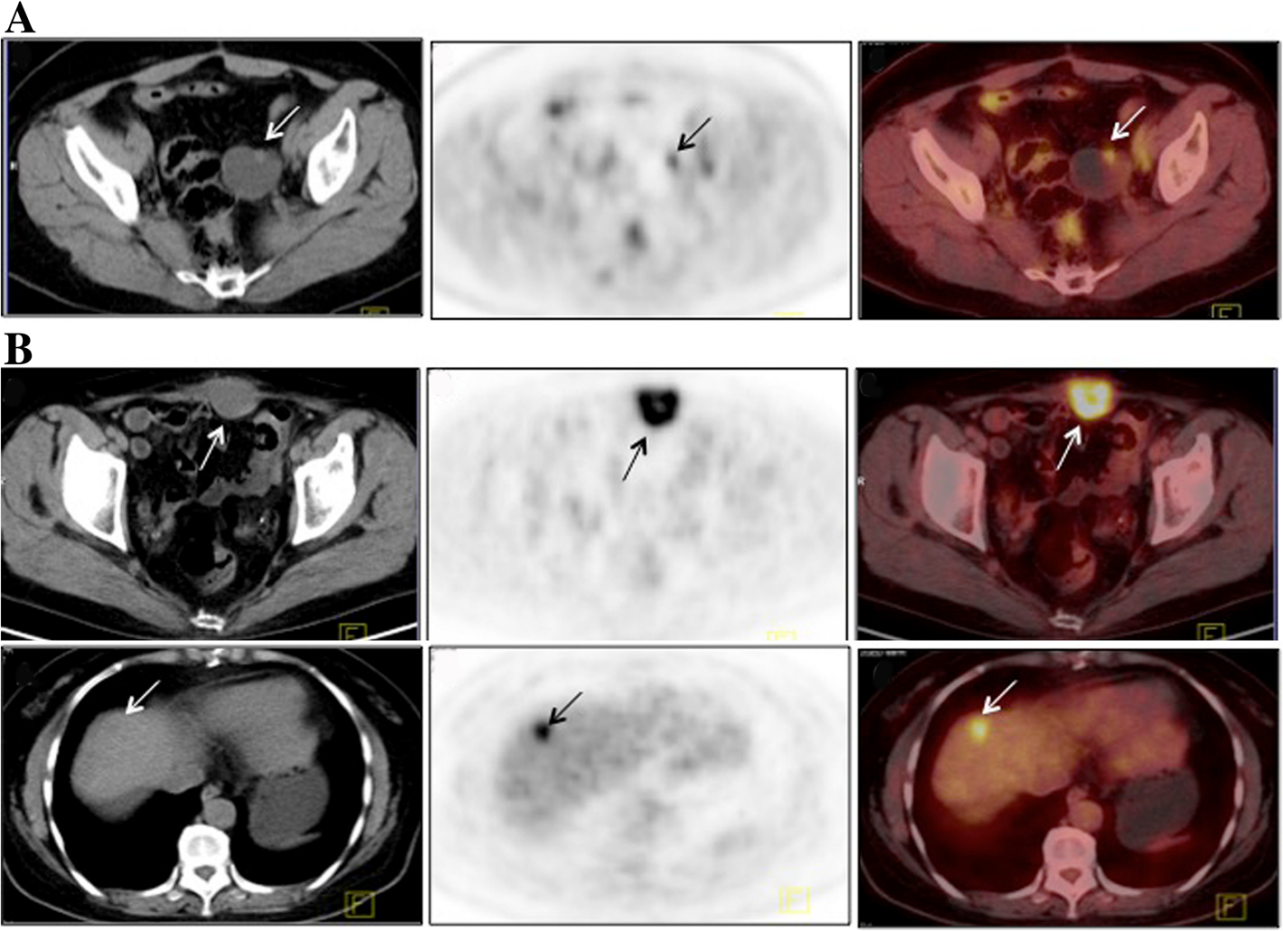
Representative PET/CT images. **a**:The patient had vaginal cuff recurrence. The SUV max, MTV60 and TLG60 was 4.1, 0.98 mL and 3. 19 g, respectively. The overall survival (OS) and post-relapse survival (PRS) was 41 and 39 months, respectively. **b**:In another patient, the recurrent tumors were located in the abdominal wall and liver. The SUV max, MTV60 and TLG60 was 13.4, 9.03 mL and 82.76g, respectively. The OS and PRS was 12 and 6 months, respectively. Abbreviations: MTV: metabolic tumor volume; TLG: Total lesion glycolysis

**Fig. 2:**
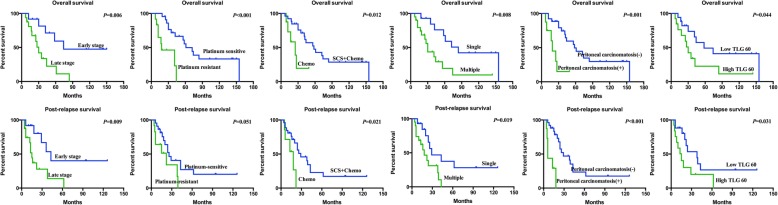
Kaplan-Meier survival curves. Abbreviations: *SCS* Secondary Cytoreduction Surgery, *Chemo* Chemotherapy, *TLG* Total lesion glycolysis

Platinum-resistant recurrence, peritoneal carcinomatosis and high TLG60 proved to be negative predicators of overall survival after multivariate analysis. In terms of post-relapse survival, only peritoneal carcinomatosis retained statistic significance on multivariate analysis. Figure 1 shows representative PET/CT images of two patients and Fig. 2 presents that patients with higher TLG60 had worse survival compared to those with lower level of TLG60.

## Discussion

The study depicted the relapse pattern of a cohort of ovarian CCC patients treated in our institution. The median PFS was 13 months (range, 2–135) for the entire population and 20 months (range, 6–108) for patients with early-stage tumor. The most commonly reported site of recurrence was pelvis (29.4%), followed by lymph node (19.6%) and peritoneal carcinomatosis (15.7%). An even higher rate (53.8%) of pelvic recurrence was noted in patients with initially early-stage disease, which supports the concept that ovarian CCC has a predilection for pelvic failure in line with previous publications [21, 22]. Importantly but not surprisingly, nearly a half of the patients had normal level of serum CA125 at recurrence.

Publications assessing the clinical utility of ^18^F-FDG PET/CT particularly in ovarian CCC patients are limited and mainly focused on patients with primary disease [23]. Ovarian CCC tumor was usually assumed to be low FDG uptake. According to a Japanese study with small cases (*n* = 11), positive FDG accumulation was shown in 54.5% patients and the median SUVmax was 3.52 before primary surgery [23]. In our previous work (under submission), we evaluated the role of preoperative PET/CT-based metabolic variables in ovarian CCC patients at first diagnosis. We found that 90% (20/22) of the patients had high-FDG tumors and the median SUVmax was 7.25. In the present study, we specifically focused on the PET/CT imaging in patients with first relapse. Again, the majority patients had FDG-avid recurrent lesions and the median SUVmax was 7.10 (range, 3.00–20.60). Further, we assessed the prognostic implications of PET/CT-based metabolic parameters and found that higher TLG60 was negative predictor for overall survival. SUVmax represented the highest SUVmax of all recurrent tumors. However, its utility in representing the metabolic activity is incomplete [12]. On the contrary, TLG might better represent the tumor burden in that it combines both volumetric and metabolic information [10]. Based on the study, we found that patients with higher level of TLG60 at first relapse had worse overall survival with statistic significance. It is noteworthy that different threshold definitions have been used in delineating metabolic tumors and no consensus has ever been achieved. In published literature, three major criteria have been employed: the absolute (SUV 2.5), relative (a fixed ratio such as 40% of SUVmax) and background-related relative thresholds (mediastinal background SUV plus two standard deviations) [12, 24]. As an exploratory study, we used two methods including absolute and relative thresholds, with the hope of finding a more appropriate threshold method in ovarian CCC patients.

According to a review article with profound influence, reprogramming energy metabolism especially glucose metabolism is considered as an emerging hallmark of cancer [25]. Quite a few publications have evaluated the clinical implication of ^18^F-FDG PET/CT based quantitative variables in ovarian cancer, irrespective of histologic subtype [10–17]. Most studies including ours focused on the prognostic implication of metabolic volumetric parameters (MTV, TLG) in ovarian cancer [10, 11, 14, 15, 17]. Besides, Vargas and colleagues investigated the predictive value of metabolic tumor volume in optimal debulking of secondary cytoreductive surgery [15]. In summary, most studies confirm that ovarian cancer patients with higher metabolic volumetric variables tended to have worse survival [10–17]. We postulated that higher metabolic volume might be a potential surrogate biomarker for disease aggressiveness and warranted more aggressiveness treatment, which requires further study.

The present study has several limitations. Firstly, selection bias might be one important problem. The cases were included by searching both the inpatient medical record system (mainly for surgery patients) and the PET/CT database in nuclear department. This is partly the reason why most patients underwent secondary surgery. Those patients who were not candidates for surgery might be missed out. Secondly, the clinicopathological information was not complete given that some patients received primary surgery at outside hospital. Thirdly, the specific details of second-line chemotherapy were not presented in the study. A part of the patients received chemotherapy in local hospital due to several reasons. Therefore, cautions should be taken when interpreting the data of study.

## Conclusion

In the study, we presented the relapse pattern of 35 ovarian CCC patients. TLG60, along with platinum-resistant recurrence and peritoneal carcinomatosis, proved to be negative predicators of overall survival after multivariate analysis. Whether patients with higher TLG60 required more aggressive treatment warranted further assessment.

## Abbreviations

^18^F-FDG PET/CT: ^18^F-fluorodeoxyglucose positron emission tomography/computed tomography
CCC: Clear cell carcinoma
MTV: Metabolic tumor volume
SUVmax: Maximum standardized uptake value
TLG: Total lesion glycolysis
FIGO: The International Federation of Gynecology and Obstetrics
PFS: Progression-free survival
OS: Overall survival
PRS: Post-relapse survival
CA: Cancer antigen
SCS: Secondary cytoreduction surgery
